# Testing the hypothesis of preferential attachment in social network formation

**DOI:** 10.1140/epjds/s13688-015-0052-2

**Published:** 2015-10-09

**Authors:** Thomas House, Jonathan M Read, Leon Danon, Matthew J Keeling

**Affiliations:** School of Mathematics, University of Manchester, Oxford Road, Manchester, M13 9PL UK; Warwick Infectious Disease Epidemiology Research (WIDER), University of Warwick, Gibbet Hill Road, Coventry, CV4 7AL UK; CHICAS, Faculty of Health and Medicine, Lancaster University, Lancaster, Lancashire LA1 4YG UK; School of Social and Community Medicine, University of Bristol, Oakfield Grove, Clifton, BS8 2BN UK

**Keywords:** MLE, Phase-type distribution, model selection, spectral methods

## Abstract

The hypothesis of preferential attachment (PA) - whereby better connected individuals make more connections - is hotly debated, particularly in the context of epidemiological networks. The simplest models of PA, for example, are incompatible with the eradication of any disease through population-level control measures such as random vaccination. Typically, evidence has been sought for the presence or absence of preferential attachment via asymptotic power-law behaviour. Here, we present a general statistical method to test directly for evidence of PA in count data and apply this to data for contacts relevant to the spread of respiratory diseases. We find that while standard methods for model selection prefer a form of PA, careful analysis of the best fitting PA models allows for a level of contact heterogeneity that in fact allows control of respiratory diseases. Our approach is based on a flexible but numerically cheap likelihood-based model that could in principle be applied to other integer data where the hypothesis of PA is of interest.

## Introduction

### Contact heterogeneity in infectious disease epidemiology

Infectious pathogens that spread via contact between people are a major cause of human disease, driving attempts to understand their epidemiology [1]. Much theoretical work on infectious disease dynamics has been focused on the role of heterogeneity in the human population [2], which is often conceptualised as a network of epidemiologically relevant contacts [3–5].

Perhaps the most important quantity in any infectious disease outbreak is the basic reproductive ratio, $R_{0}$, which is defined verbally as the expected number of secondary cases generated by an average primary case early in the epidemic. An epidemic is possible exactly when $R_{0}>1$, and typically the efforts required to control such an outbreak grow monotonically with $R_{0}$ [1, 2]. In the simplified scenario where each individual picks an integer number of contacts *K* from the same *degree distribution*, but transmission is otherwise homogeneous,

1 $$ R_{0} \propto\mathbf{E}\bigl[K^{2}\bigr] . $$

This dependence of the basic reproductive ratio on the second moment of the degree distribution has been a ‘textbook’ result for some time [6], however work by Pastor-Satorras and Vespignani [7] and May and Lloyd [8] raised the question of what might happen for large, or asymptotically divergent, second moments. Such questions can be posed and answered at different levels of mathematical rigour [9] however in the context of epidemiology it is clear that a highly variable degree distribution is associated with the epidemiologically unrealistic scenario that even the most weakly transmissible pathogen can cause an epidemic, and as a corollary that control of any infectious disease through untargeted vaccination would be impossible.

### Data

Of course, whether such a theoretical possibility matters for the study of infectious diseases depends on the actual variance in degree for epidemiologically relevant contacts. While 20th century models of infectious disease were often based on strong a priori assumptions about mixing patterns [1], various methods for measurement of contact patterns now exist and were reviewed by Read et al. [10]. As well as direct measurement of individuals through surveys [11] it is possible to improve coverage through snowball and respondent-driven sampling [12, 13], to make use of the extremely large datasets produced by electronic sensors [14, 15], and also to combine aggregate data [16, 17].

Previous empirical studies have seen evidence that for direct (e.g. [16, 18]) and sexual (e.g. [19, 20]) contacts, an approximate power-law relationship may hold such that for large *k*, a randomly selected node obeys

2 $$ \operatorname{Pr}(\text{node has } k \text{ links}) \approx k^{-\gamma} . $$

As is the case for almost all biological data, there is much more complexity in the data than such a simple parametric relationships as (2) would imply. For example, Leigh Brown et al. [20] found that while a power-law was a better functional form than the negative binomial for sexual contacts, the richer Waring distribution was preferable to either. What is hard to dispute, however, is that as better quality data on epidemiologically relevant contacts is obtained the evidence consistently points to a very high level of variance; for example, Read et al. [21] were able to validate the high numbers of contacts reported by some study participants through direct (rather than postal) surveying.

These empirical observations of high heterogeneity in contact number, together with theoretical results about $R_{0}$, present a paradox for infectious disease epidemiology: is the extreme heterogeneity in observed contact patterns indicative of PA and does that imply that $R_{0}>1$ for almost any finite level of person-to-person transmissibility meaning that our theoretical understanding of infectious disease epidemiology is somehow severely lacking?

### Preferential attachment and power laws in empirical data

Recent years have seen a debate about the level of heterogeneity that exists in a variety of observed networks. A particularly influential paper by Barabási and Albert [22] considered a model of network formation in which many new nodes are added to a small existing network. These new nodes connect preferentially to nodes that have more links in the existing network, leading to the asymptotic result (2) with $\gamma=3$. In this way preferential attachment is intimately linked with, but not always equivalent to, asymptotic power-law behaviour.

Simple power-law relationships have been claimed for numerous real-world systems, and a critical review of these claims by Clauset et al. [23] used maximum-likelihood fitting of distribution tails to power-law distributions to show varying levels of statistical support for claims in the literature. In the context of discrete data, pioneering work by Zipf [24] found power-laws in word frequencies; considering the count of unique words in *Moby Dick* both Newman [25] and Clauset et al. [23] agree that the statistical evidence for Zipf’s power-law distribution in this context is strong. On the other hand, the in- and out-degrees of *E. coli* metabolic networks have been claimed to follow a power law [26], but this is disputed by the analyses of Huss and Holme [27] and Clauset et al. [23].

The debate around presence or absence of power laws in real data continues, perhaps most strongly in the context of networks. For example, Barabási [28] writes that preferential attachment is network science’s “most profuse concept,” and that “the impact of preferential attachment is hard to miss.” At the same time, Stumpf and Porter [29] argue that “most reported power laws lack statistical support and mechanistic backing.”

### Testing preferential attachment directly

In this work, we attempt to test the hypothesis of preferential attachment in social contact data directly, rather than via asymptotic power law behaviour. We make use of previously collected data on social encounters specifically designed to measure heterogeneity in numbers of contacts amongst the British population, and fit mechanistic models of different complexity to these data. We determine that models with significant levels of preferential attachment have better evidential support from the data than models without.

## Methods

### Social Contact Survey data

A cross-sectional study was conducted between May 2009 and October 2010, recruiting households and individuals through postal and online questionnaires, supported by a large random-address mailshot and a modest online and media promotion [30, 31]. Questionnaires asked respondents to report on the number of distinct individuals they encountered the previous day: their contacts. Respondents were able to report contacts either as individuals or as members of a group with a reported size. Allowing the reporting of groups of individuals was a deliberate methodological design to permit the easy reporting of large numbers of contacts, to avoid the approach taken by previous studies [11], which imposed a high burden on respondents with large number of contacts, and to ensure the best capture of the right-hand tail of the degree distribution. In general, we expect that such data will become increasingly available due to the epidemiological importance of this tail (e.g. the study of Read et al. [21]).

In total, completed questionnaires were received from 5,388 participants in Great Britain, 3,901 of which were from postal surveys. There was some bias in demographical representation, most notably younger age groups and males were generally under-represented (see Danon et al. [31] for more details). The data is available at http://wrap.warwick.ac.uk/54273/.

### Generalised preferential attachment

As noted by Durrett [32], Barabási [28], and Simkin and Roychowdhury [33], the basic idea behind the preferential attachment model is close to the population model of Yule [34]. We consider a Yule-like stochastic process described precisely as follows. In a population of *N* individuals indexed by *i* each individual has an integer-valued random variable $K_{i}(t)$ for its number of contacts. Individual *i* starts with $K_{i}(0)=0$ and makes new contacts over a time period $T_{i}$, which is given by a positive real-valued random variable with probability density function $\rho(t)$. The generation of new social contacts is modelled by a continuous-time Markov chain with the following events and rates:

3 $$ K_{i} \rightarrow K_{i} + 1 \quad\text{at rate } f_{K_{i}} := 1 + \tau K_{i} . $$

We take the preferential attachment hypothesis PA to be stated mathematically as

4 $$ \text{PA} \Leftrightarrow\tau>0 . $$

Writing $p_{k}(t)$ for the probability that $K_{i}=k$ at time $t< T_{i}$, we can use the method of characteristics to derive an expression for the probability generating function of $K_{i}$,

5 $$ g(t,s) = \sum_{k} p_{k}(t) s^{k} = \bigl(s - (s-1) \mathrm{e}^{\tau t} \bigr)^{-1/\tau} ,\quad s \in[0,1] . $$

From this, we can derive expressions for the probability mass function,

6 $$\begin{aligned} p_{k}(t) =& \frac{1}{k!} \frac{\partial^{k} g}{\partial s^{k}} \bigg|_{s=0} = \frac{\Gamma(k+\frac{1}{\tau})}{\tau\Gamma(\frac{1}{\tau })\Gamma(k+1)} \mathrm{e}^{-t}\bigl( \mathrm{e}^{\tau t} - 1\bigr)^{k} \\ \rightarrow& \kappa_{t} \bigl(\mathrm{e}^{\tau t} - 1 \bigr)^{k} k^{{(1-\tau)}/{\tau}} , \end{aligned}$$

where $\kappa_{t}$ is a function of *t* but not *k* and the asymptote holds as *k* becomes large. This is not a simple power-law relationship, and so the asymptotic behaviour of the moments is not determined by the power-law exponent, but rather through the moment generating function $M(t,z) = g(t,\mathrm{e}^{z})$, $z\in(-\infty,0]$, such that the *r*th moment of the degree distribution, conditional on $t< T$, is

7 $$ m_{r}(t) = \frac{\partial^{r} M}{\partial z^{r}} \bigg|_{z=0} . $$

In particular,

8 $$ m_{1}(t) = \frac{1}{\tau}\bigl(\mathrm{e}^{\tau t} -1\bigr) , \quad\quad m_{2}(t) = m_{1}(t) + (\tau+1) \bigl(m_{1}(t)\bigr)^{2} ,\quad\quad \ldots. $$

Then accounting for the randomness of the times, the *r*th moment of the degree distribution will be

9 $$ \overline{m}_{r} = \int_{t=0}^{\infty} \rho(t) m_{r}(t)\,\mathrm{d}t . $$

We will be interested in the empirical evidence for whether such moments converge or diverge, in light of the epidemiological relationship (1).

### Phase-type holding times

The question is then posed as to an appropriate distribution from which to draw the holding times $\{T_{i}\}$ for the amount of time spent making new contacts on the day for which individuals provide data. In previous work [30] on a related model of contact formation we considered holding times $T_{i}$ that were log-normally distributed. This provided a good fit to data, but was computationally intensive and lacked a mechanistic interpretation. We therefore consider here a class of distributions for the holding times that is highly flexible, but which has analytic and numerical benefits - the distributions of *phase type* [35]. Phase-type distributions are dense in the space of positive-valued probability distributions [36], meaning that they can be made arbitrarily close to any other distribution. They have a mechanistic interpretation and allow for analytic manipulations that greatly reduce the numerical cost of likelihood evaluation.

**Figure 1:**
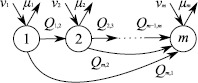
**A model of** ***m*** **phases.** $\nu_{a}$ is the probability of starting in phase *a*, $\mu_{a}$ is the exit rate of phase *a*, and $Q_{a,b}$ is the rate of moving from phase *a* to phase *b*.

The basic idea behind the model is shown in Figure 1. A set of phases is indexed by $a,b=1,\ldots, m$; the probability of starting in phase *a* is $\nu_{a}$ (meaning these parameters must sum to unity); the rate of stopping making new social contacts is $\mu_{a}$ for an individual in phase *a*; and the rate of moving from phase *a* to phase *b* is $Q_{a,b}$. Note that different rates of making contacts in different phases are not realistically distinguishable from different times spent and so are not included as parameters. The phases have a mechanistic interpretation as the activities that individuals undertake on a given day.

In this model, the probability density function for the holding time is given by the general expression

10 $$ \rho(t) = \boldsymbol{\mu}^{\top} \mathrm{e}^{\mathbf{M} t} \boldsymbol{\nu} , $$

where:

11 $$ \begin{aligned} &\boldsymbol{\mu} = (\mu_{a});\quad\quad \boldsymbol{\nu} = (\nu_{a}) ; \\ & M_{a,a} = -M_{a} ;\quad\quad M_{a} =\mu_{a} + \sum_{b} Q_{b,a} ;\quad\quad M_{a,b\neq a} = Q_{b,a} . \end{aligned} $$

From the expressions (5), (7), (8) and (9) above, in particular through inspection of the form of the moment generating function, it is clear that the *r*th moment of the degree distribution will involve a term like

12 $$ \mathcal{I}_{r} = \int_{t=0}^{\infty} \mathrm{e}^{r\tau t} \rho(t) \,\mathrm{d}t = \boldsymbol{\mu}^{\top} \int_{t=0}^{\infty} \mathrm{e}^{(\mathbf{M} + r\tau\mathbf{I})t} \,\mathrm{d}t \boldsymbol {\nu} , $$

where **I** is the identity matrix. Let $\mathbf{A} = \mathbf {M} + r\tau \mathbf{I}$; this matrix is triangular and so its eigenvalues are equal to its diagonal elements; in particular the *a*th eigenvalue of **A** is $\lambda_{a} = -M_{a} + r \tau$. If we let **R** be a matrix whose *a*th column is the *a*th right eigenvector of **A** and **L** be a matrix whose *a*th row is the *a*th left eigenvector of **A** then

13 $$ \mathcal{I}_{r} = \boldsymbol{\mu}^{\top} \int _{t=0}^{\infty} \mathbf{L}^{-1} \mathbf{L} \mathrm{e}^{\mathbf{A}t} \mathbf{R} \mathbf{R}^{-1} \,\mathrm{d}t \boldsymbol{\nu} = \boldsymbol{\mu}^{\top} \mathbf{L}^{-1} \int _{t=0}^{\infty} \mathbf{D} \,\mathrm{d}t \mathbf{R}^{-1}\boldsymbol{\nu} , $$

where **D** is a diagonal matrix whose *a*th diagonal element is $\mathrm{e}^{\lambda_{a} t}$. The integral $\mathcal{I}_{r}$ therefore converges exactly when ∀*a*, $\lambda_{a}<0$, which implies that the condition for divergence of the *r*th moment is

14 $$ \overline{m}_{r} \text{ diverges}\quad \Leftrightarrow\quad \exists a \text{ such that } \tau> M_{a}/r . $$

In general, however, combination of (10) and (6) is not the most numerically efficient method for calculation of the overall probability mass function for final number of contacts $K_{i}(T_{i})$ and a different approach is needed.

### Numerically efficient model solution

The model as described above can be solved in a numerically efficient manner using the spectral methods for continuous-time Markov chains developed by Bailey [37]. We consider the limit as the population size $N\rightarrow\infty$ and write down ordinary differential equations (ODEs) for the proportion of the population in phase *a* and with *k* social contacts at time *t*, $p_{a,k}(t)$. These ODEs take the form

15 $$ \frac{\mathrm{d}}{\mathrm{d}t}p_{a,k} = - \biggl(f_{k} + \mu_{a} + \sum_{b>a}Q_{a,b} \biggr) p_{a,k} + f_{k-1} p_{a,k-1} + \sum _{b< a} Q_{b,a}p_{b,k} , $$

where $f_{k}$ is the rate at which individuals with *k* social contacts make new contacts, given in (3). We are then interested in $d_{k}$, the probability mass function for a randomly selected individual having made *k* social contacts by the end of the process. A series of manipulations directly analogous to those of Bailey [37] shows that

16 $$ d_{k} = \lim_{s\downarrow0} \sum _{a} \mu_{a} \int_{0}^{\infty} \mathrm{e}^{-st} {p}_{a,k}(t)\, \mathrm{d}t =: \sum _{a} \mu_{a} A_{a,k} . $$

Applying Laplace transformation to (15) subject to the initial condition $p_{a,k}(0) = \nu_{a} \delta_{k,0}$ and taking the frequency-space limit $s\downarrow0$ then leads to a set of linear equations for $d_{k}$ that are triangular and so can be evaluated directly without numerically costly matrix inversion:

17 $$ \nu_{a} \delta_{k,0} = - \biggl(f_{k} +\mu_{a} + \sum_{b>a}Q_{a,b} \biggr) A_{a,k} + f_{k-1} A_{a,k-1} + \sum _{b< a} Q_{b,a}A_{b,k} . $$

These equations are at the root of the numerical efficiency of our model. Note that we use Laplace transformation mainly for technical reasons and our results could be obtained by directly integrating (15) if one were not concerned by all quantities being rigorously defined.

### Model likelihood, fitting and selection

We assume a vector of data $\mathbf{y}=(y_{k})$, where $y_{k}$ is the number of individuals reporting *k* social contacts when surveyed. A model $\mathcal{M}$ is therefore specified by a number of phases *m* and the presence or absence of PA, meaning the general parameters are $\boldsymbol{\theta} = (\tau,\nu_{a},\mu_{a},Q_{a,b})$, with *τ* present only if there is PA. The number *n* of individuals sampled from the British population *N* is

18 $$ n = \sum_{k} y_{k} = 5388 \ll N \gtrsim6 \times10^{7} , $$

and so it is appropriate to assume that each individual picks a number of contacts independently from the distribution with pmf given by $d_{k}$ as in (16). Accounting for the censoring of zero contacts in the real data, we define

19 $$ \tilde{d}_{0} = 0 ,\quad\quad \tilde{d}_{k>0} = \frac{d_{k}}{1-d_{0}} , $$

meaning that the overall likelihood function is then given by

20 $$ L(\mathbf{y} | \boldsymbol{\theta}) = \frac{n!}{\prod_{l} y_{l} !} \prod _{k} \bigl(\tilde{d}_{k}(\boldsymbol{ \theta})\bigr)^{y_{k}} . $$

Note that the combinatorial factors do not depend on the parameters, and so need not be calculated during model fitting.

We consider the use of the likelihood function (20) using standard statistical methodology. Numerical maximum likelihood estimation was performed using simulated annealing run from multiple starting points to ensure the global optimum was obtained. Model selection was performed using AIC [38] and BIC [39], as well as likelihood ratio tests [40] on pairs of models where this test was informative. This was done since each approach involves different trade-offs between model fit and complexity, and to check that our conclusions about PA are not overly sensitive to the precise method used. Uncertainty in model parameters was quantified using confidence intervals obtained through bootstrapping the data, and uncertainty in model outputs such as the predicted degree distribution was quantified using a parametric bootstrap.

## Results and discussion

**Figure 2:**
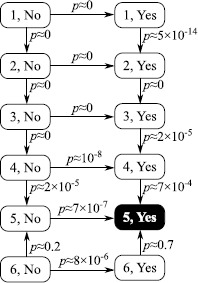
**The results of likelihood ratio tests on the models.** Arrows point towards the model preferred by the likelihood ratio test, with *p* values shown.

Table 1 shows the models we fitted, their number of parameters, AIC/BIC relative to the minimum, and the first moment that diverges according to (14). Figure 2 shows the results of performing likelihood ratio tests. These show that AIC prefers a 5-phase model with PA as do likelihood ratio tests for any significance level between 0.07% and 20%. BIC penalises complex models more severely and therefore selects a 3-phase model with PA.

**Table 1:** **Comparison of models with different numbers of phases, with and without preferential attachment (PA), together with: number of parameters; differences in AIC and BIC values compared to the overall minimum; and the lowest divergent moment for models with PA**

**(Phases, PA)**	**No. Params**	**ΔAIC**	**ΔBIC**	**Diverge**
(1,No)	1	2.2 × 10^3^	2.1 × 10^3^	–
(2,No)	4	2.1 × 10^2^	1.5 × 10^2^	–
(3,No)	8	1.2 × 10^2^	83	–
(4,No)	13	42	38	–
(5,No)	19	23	58	–
(6,No)	26	27	1.1 × 10^2^	–
(1,Yes)	2	1.9 × 10^2^	1.1 × 10^2^	3
(2,Yes)	5	1.3 × 10^2^	72	4
(3,Yes)	9	31	$\mathbf{[0]}$	3
(4,Yes)	14	11	14	3
(5,Yes)	20	$\mathbf{[0]}$	42	3
(6,Yes)	27	9	97	3

Preferred models are shown using square brackets and bold type.

**Figure 3:**
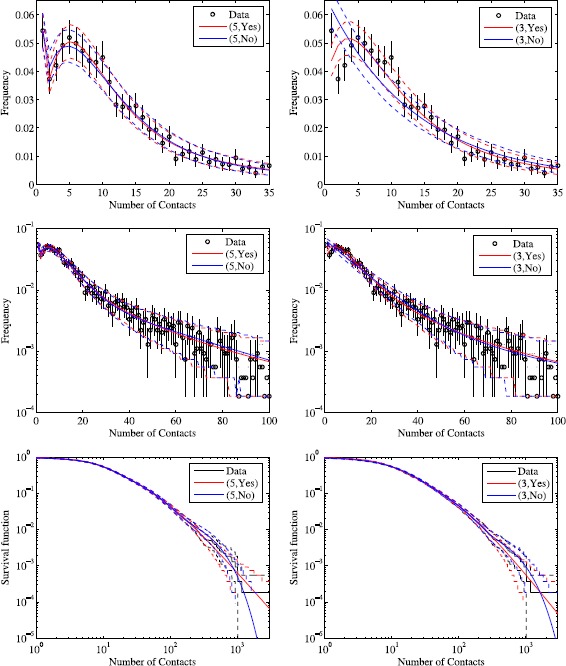
**Data at different scales versus (left column) model selected using AIC and likelihood ratio tests (right column) model selected using BIC.** Models are labelled by the number of phases and whether PA is present. Confidence intervals in the data are calculated using bootstrapping for data and parametric bootstrapping for models.

Therefore, regardless of the number of phases selected by different approaches to model selection, we see that the models with PA are preferred over models without. Figure 3 shows the predictions of the models preferred by different selection criteria, as well as the models with the same number of phases but no PA, against real data. We see in the left column of plots that for the 5-phase models, the main difference is in the tail of the distribution as we would expect. In the 3-phase models shown in the right column of plots, the model without PA also smooths over features in the bulk of the distribution compared to the model with PA.

For the 3-phase model with PA, $\tau= 0.018\ [0.012,0.026]$; and if we set $\tau=0$ but leave the other parameters at their fitted values, then the total number of contacts per person is reduced to 64% of its original value. For the 5-phase model with PA $\tau= 0.026\ [0.019,0.036]$; and if we set $\tau=0$ but leave the other parameters at their fitted values, then the total number of contacts per person is reduced to 58% of its original value. This shows that in both of these models, we can attribute a substantial fraction of the contacts to PA.

We also calculate that the second moment does not diverge in any of the fitted models, which helps to resolve the epidemiological paradox that we introduced at the start of this paper. PA is empirically supported, and is also mechanistically plausible since existing social contacts give more opportunities for future social contact. Combined with a sufficiently detailed phase-based mechanistic model of the contexts in which social contacts are made, however, PA does not imply a divergent second moment for the distribution of contacts relevant for the spread of directly transmitted infections. This means that our understanding of how basic epidemiological quantities like the basic reproductive ratio, $R_{0}$, are related to contact networks does not need to be revised in the light of empirical evidence.

As a final observation, we believe that as computational resources for fitting models to data improve, it will in general be easier to test the hypothesis of PA directly in all kinds of data, rather than looking for asymptotic power laws.

## Appendix 1: Code

We provide the function phase_ll.c below as a Mex file for use within MATLAB; however, the C syntax is standard and modification to run within R, Python or C should be straightforward. Once compiled, use within MATLAB is:

>> [LL, P] = phase_ll(params,M,kh,K);

where params is a vector of the model parameters [nu(:), mu(:), Q(:), tau], M is the number of phases, kh is a vector of count frequencies from 0:K, K is the maximum count, LL is the log-likelihood given the parameters and P is a the probability vector for the observed counts given the parameters.

### 1.1 The function phase_ll.c

#include <mex.h>

#include <math.h>

void mexFunction (int nlhs, mxArray *plhs[],

                  int nrhs, const mxArray *prhs[])

{

  /* Input variables */

  double *params, *kh, *Mmex, *Kmex;

  size_t lp;

  int M, K;

  /* Get inputs in correct form */

  params = mxGetPr(prhs[0]);

  lp = mxGetN(prhs[0]);

  Mmex = mxGetPr(prhs[1]);

  kh = mxGetPr(prhs[2]);

  Kmex = mxGetPr(prhs[3]);

  M = (int) Mmex[0];

  K = (int) Kmex[0];

  /* Working variables */

  double nu[M], mu[M], Q[M][M], s[M];

  double f[K];

  double tau, S;

  double A[K][M];

  int fi, fj, k, m;

  /* Output variables */

  double *P, *LL;

  /* Organise outputs into correct form */

  plhs[0] = mxCreateDoubleMatrix(1,1,mxREAL);

  LL = mxGetPr(plhs[0]);

  plhs[1] = mxCreateDoubleMatrix(K,1,mxREAL);

  P = mxGetPr(plhs[1]);

  nu[0] = 1.0;

  for(fi=1; fi<M; fi++) {

    nu[fi] = params[fi-1];

    nu[0] -= params[fi-1];

  }

  for(fi=M; fi<(2*M); fi++) {

    mu[fi-M] = params[fi-1];

  }

  m = (2*M)-1;

  for(fi=0; fi<M; fi++) {

    s[fi] = 0;

    for(fj=(fi+1); fj<M; fj++) {

      Q[fi][fj] = params[m];

      s[fi] += params[m];

      m=m+1;

    }

  }

  if (lp == (m+1))

    tau = params[m];

  else

    tau = 0;

  f[0] = 1;

  for(k=1; k<(K-1); k++)

    f[k] = 1+(tau*k);

  f[K-1] = 0;

  A[0][0] = (nu[0])/(f[0]+mu[0]+s[0]);

  for(k=1; k<K; k++) {

    A[k][0] = ( f[k-1]/(f[k]+mu[0]+s[0]) )*A[k-1][0];

  }

  for(m=1; m<M; m++) {

    S=0.0;

    for(fi=0; fi<m; fi++) {

      S += Q[fi][m]*A[0][fi];

    }

    A[0][m] = (nu[m]+S)/(f[0]+mu[m]+s[m]);

    for(k=1; k<K; k++) {

      S=0.0;

      for(fi=0; fi<m; fi++) {

        S += Q[fi][m]*A[k][fi];

      }

      A[k][m] = ((f[k-1]*A[k-1][m])+S)/(f[k]+mu[m]+s[m]);

    }

  }

  P[0] = 0.0;

  for(m=0; m<M; m++) {

    P[0] += A[0][m]*mu[m];

  }

  for(k=1; k<K; k++) {

    P[k] = 0.0;

    for(m=0; m<M; m++) {

      P[k] += A[k][m]*mu[m];

    }

    P[k] /= (1-P[0]);

  }

  P[0] = 0.0;

  LL[0] = 0.0;

  for(k=1; k<(K-1); k++) {

    LL[0] += kh[k]*log(P[k]);

  }

}
